# Editorial: Current evidence on epidemiology and management of infections in critically ill patients

**DOI:** 10.3389/fpubh.2025.1584879

**Published:** 2025-06-10

**Authors:** Francisco Eduardo Dardón-Fierro, Ricard Ferrer, Erika P. Plata-Menchaca

**Affiliations:** ^1^Intensive Care Unit, Bellvitge University Hospital, Barcelona, Spain; ^2^Intensive Care Unit, Shock, Organ Dysfunction and Resuscitation Group, Vall d'Hebron Research Institute, Vall d'Hebron Hospital Campus, Barcelona, Spain; ^3^Intensive Care Unit, Hospital Clinic, Barcelona, Spain

**Keywords:** sepsis, COVID-19, communicable diseases, shock, septic, critical care, drug resistance

Critically ill patients face heightened risks of infections due to immunosuppression, invasive procedures, and prolonged intensive care unit (ICU) stays. These infections, particularly those caused by multidrug-resistant (MDR) pathogens, remain a leading cause of morbidity and mortality globally, with evolving challenges such as antimicrobial resistance (AMR), diagnostic limitations, and emergent pathogens like SARS-CoV-2 exacerbating outcomes. The COVID-19 pandemic further underscored these vulnerabilities, revealing how overwhelmed healthcare systems and immunosuppressive therapies amplify secondary infections (1–5).

The rationale for this Research Topic (RT) covers the following points:

1. Epidemiological shifts: up to 54% of ICU patients develop nosocomial infections, with bloodstream infections (BSIs), ventilator-associated pneumonia (VAP), and fungal infections driving poor prognoses (1–3, 5).
2. AMR crisis: MDR Gram-negative bacteria (e.g., Pseudomonas aeruginosa, Acinetobacter spp.) and fungi increasingly complicate management, with resistance rates exceeding 60% in some cohorts (2–4).
3. Diagnostic and therapeutic gaps: traditional tools like blood cultures and qSOFA scores lack sensitivity, delaying targeted therapy (6, 7). Simultaneously, balancing antimicrobial stewardship with effective treatment remains contentious (8–11).

The aim of this RT is to address and synthesize recent advances and persisting challenges in infection management for critically ill patients, with three key aims:

1. Analyze evolving epidemiology of ICU-acquired infections, including post-COVID-19 trends.
2. Evaluate innovations in diagnostics (e.g., genomic sequencing) and personalized treatment strategies (e.g., procalcitonin-guided de-escalation, immunomodulation).
3. Highlight actionable frameworks for optimizing antimicrobial use, sepsis management, and infection prevention in resource-limited settings.

As we know, infections in critically ill patients encompass a wide spectrum of clinical conditions, ranging from the early identification of infections associated with a high risk of sepsis, to septic shock and multiple organ dysfunction. These conditions continue to present major challenges to critical care clinicians. The continuous emergence of MDR bacterial infections in the general population of critically ill patients further complicates the diagnosis and management of infections. Some studies have identified MDR organisms in up to 61% of critically ill patients (12). In this regard, two studies examined the prevalence and impact of MDR infections in critically ill patients. In a retrospective study, Zhang et al. analyzed a total of 372 children diagnosed with carbapenem-resistant (CRAB) and carbapenem-sensitive (CSAB) *Acinetobacter baumanii* at a pediatric intensive care unit (PICU) in a tertiary hospital in China from 2016 to 2021. The primary risk factors associated with CRAB were recent invasive procedures, gastric intubation, prior carbapenem use, severe pneumonia and anemia with hemoglobin values of < 110 g/L. Significant factors associated with overall mortality were septic shock, AST levels >46 U/L, bone marrow aspiration, lymphocyte count < 20% and advanced age. In Brazil, Martins et al. studied 757 patients admitted to the intensive care unit (ICU) of a government university hospital from 2018 to 2020. They found a high prevalence of MDR organisms in critically ill patients (~9.5%), which highlights the significant threat posed by these infections in ICUs.

Early sepsis diagnosis and management are often insufficiently performed. The initial symptoms of sepsis are non-specific, making early diagnosis challenging. The current diagnostic criteria for sepsis screening, such as the quick sequential organ failure assessment (qSOFA) criteria, have limited sensitivity, excluding more than half of sepsis cases. In addition, blood cultures, which are considered the gold standard for infection detection, are negative in around 50% of sepsis cases (7). This diagnostic uncertainty frequently leads to delays in delivering appropriate treatment. In a single-center study, Liu et al. analyzed the prevalence of pneumonia-related bloodstream infections (PRBSI) in a cohort of critically ill patients from 2017 to 2020. Using whole genome sequencing to enhance bacterial identification, they confirmed bloodstream infections (BSI) in 120 patients. PRBSI was documented in 26% of patients. Compared to non-PRBSI patients, those with PRBSI had higher 28-day mortality (81.3 vs. 51.1%), overall mortality (93.8 vs. 64.8%), longer durations of mechanical ventilation (16 vs. 6 days), and longer ICU stays (21 vs. 10 days). These findings underscore the impact of the primary origin of BSI on patient outcomes.

The rapid emergence of severe acute respiratory syndrome coronavirus 2 (SARS-CoV-2) led to a global surge of critically ill patients, many presenting with acute respiratory distress syndrome (ARDS), secondary bacterial or fungal infections, and a high risk of thromboembolic complications (13). Effective management of severe COVID-19 cases demanded an increasing understanding of the pathophysiology of the disease, characterized by a dysregulated immune response, hypercoagulability, and direct viral-mediated tissue and endothelial damage (14).

Recognizing these mechanisms was pivotal in guiding therapeutic interventions, including the judicious use of low-dose corticosteroids, antithrombotic treatment, and immunomodulatory agents. Emerging paradigms for optimizing non-invasive respiratory support, mechanical ventilation and prone positioning have been extensively studied (15, 16). Clinical biomarkers were also identified as useful tools for patient classification and prognostication. In a retrospective study, Kaddoura et al. analyzed the prevalence of hypertriglyceridemia in patients admitted to the ICU due to severe SARS-CoV-2 infection. Notably, 82.3% of the patients exhibited hypertriglyceridemia, which was associated with longer recovery times and increased ICU and hospital stays. No significant differences in mortality were observed. Interestingly, 33.8% of patients with hypertriglyceridemia received treatment with fibrates or omega-3 supplements which, despite having beneficial impact in other settings, were associated with a worse prognosis.

Best practice standards on the management of sepsis and septic shock require a holistic approach involving the timely delivery of treatment pillars, such as appropriate antimicrobial therapy, source control, rational fluid resuscitation, and early vasopressor support (17). However, the best strategies for fluid management and vasopressor use remain subjects of ongoing debate. The complexity of sepsis pathophysiology and the heterogeneity of patient responses has recently highlighted the need for more personalized approaches to sepsis diagnosis and treatment.

Ongoing research into early and prognostic biomarkers at the bedside, genetic signatures, and immune responses recognition systems hold promise for improving early diagnosis of sepsis, and tailoring treatments to individual patient phenotypes. The study of adjunctive therapies, such as hemadsorption and immunomodulation has improved in recent years due to better patient selection and predictive enrichment in clinical trials. Some reports suggest these therapies may benefit certain subgroups of patients, although further research is necessary to define their role in sepsis management (12) (Chiscano-Camón et al.). In this context, Chiscano-Camón et al. published a comprehensive review on the current perspective in sepsis management, analyzing the benefits of phenotyping sepsis patients and reviewing the state of the art in standard and adjunctive therapies.

The wide spectrum of presentation and severity of infections in critically ill patients involves new paradigms on the diagnosis and management of organ-specific infections with high-risk of sepsis, and the implementation of early interventions in sepsis and septic shock. Evolving evidence on epidemiology, risk factors, biomarkers, fenotyping and precision medicine is already contributing to improved patient outcomes. Ongoing discoveries are being evaluated to assist clinicians in diagnosing sepsis earlier, before overt irreversible organ dysfunction becomes evident through clinical scores, thereby preventing disease progression and further clinical deterioration.
